# WHOM DO WE SERVE? DIVERSITY OF OLDER COMMUNITY CARE RECIPIENTS’ FUNCTIONING ACROSS EUROPE

**DOI:** 10.1093/geroni/igz038.142

**Published:** 2019-11-08

**Authors:** Annerieke Stoop, Manon Lette, Simone de Bruin, Giel Nijpels, Hein van Hout

**Affiliations:** 1 National Institute for Public Health and the Environment, Bilthoven, Netherlands; 2 Amsterdam UMC, Amsterdam, Netherlands

## Abstract

Across Europe, an increasing number of older people with multiple health and social care needs stay in their own homes until old age. Community care aims to support them to live at home for as long as possible. Comparative studies showed that population characteristics of older community care recipients differ between European countries. This is due to differences in financing, delivery and governance of community care. However, little is known about differences in health, including physical, cognitive, mental and social functioning, of older community care recipients served across European countries. The aim of this study was to provide insight into these differences. We used data of the IBenC study, which was collected using the interRAI HC-Assessment among 2884 older community care recipients from six European countries: Belgium, Finland, Germany, Iceland, Italy and the Netherlands. We found that prevalences of impairments in different health domains were highest among Italian community care recipients followed by the Belgian population, and lowest among community care recipients from the Netherlands. Feelings of loneliness were lowest among the Italian and highest among the Dutch population. This variation between European countries may be explained by differences in eligibility for and access to formal community services and informal care provision as well as cultural diversity. Insight in these differences supports understanding of community care across Europe among European and national policy-makers and researchers.

